# Knowledge and attitude towards antimicrobial resistance among final year undergraduate paramedical students at University of Gondar, Ethiopia

**DOI:** 10.1186/s12879-018-3199-1

**Published:** 2018-07-06

**Authors:** Mohammed Assen Seid, Mohammed Seid Hussen

**Affiliations:** 1 Department of Clinical Pharmacy, College of Medicine and Health Sciences, University of Gondar, Gondar, Ethiopia; 2 Department of Optometry, College of Medicine and Health Sciences, University of Gondar, Gondar, Ethiopia

**Keywords:** Antimicrobial resistance, Knowledge, Paramedical students, Ethiopia

## Abstract

**Background:**

Globally, antimicrobial resistance (AMR) is a complex public problem, which is mainly fuelled by inappropriate use of antimicrobials. Rational use of antimicrobials is the main strategy for the prevention of AMR, which can be achieved by changing the prescribers’ behavior and knowledge. Hence, this study aimed to assess knowledge and attitude of paramedical students regarding antimicrobial resistance, which helps to rationalize the use of antimicrobials.

**Methods:**

An institutional based cross-sectional study was performed on 323 graduates paramedical students at the University of Gondar, Ethiopia. Participants were invited to complete a self-reported structured questionnaire on hard copy. The data were summarized using summary statistics such as the median. Furthermore, Kruskal Wallis test, at the level of significance of 0.05, was conducted to compare group difference.

**Results:**

Among 360 eligible paramedical students, 323 (90%) of them participated and most of them were males 202 (62.5%). Nearly 96% of the participants perceived that antimicrobial resistance is a catastrophic and preventable public problem but about half of the participants (55%) had a poor level of knowledge. It was also found that there was a statistically significant knowledge and attitude difference across the department (*p*-value< 0.0001) and (*p* = 0.002), respectively. Furthermore, those participants who had a good level of knowledge had greater attitude rank as compared to those who had a moderate and poor level of knowledge (*p*-value< 0.0001).

**Conclusion:**

Majority of the participants viewed antimicrobial resistance as a preventable public problem if appropriate strategies are formulated. Nonetheless, most of them had a poor knowledge regarding antimicrobial resistance, and their knowledge and attitude significantly vary across their field of study. This result implicates that improving the students’ level of knowledge about antimicrobial resistance might be an approach to flourish their attitude and to rationalize their antimicrobial use.

**Electronic supplementary material:**

The online version of this article (10.1186/s12879-018-3199-1) contains supplementary material, which is available to authorized users.

## Background

Nowadays, antimicrobial resistance (AMR) is a complex global major public health challenge, particularly in developing countries. It poses a catastrophic threat to the effective treatment of an ever-increasing range of infectious disease [1–3]. AMR results in reduced drugs’ efficacy, making the treatment of patients difficult, costly, or even impossible. Ultimately, it ends up with prolonged illness and increased mortality [3].

The development of AMR is a natural phenomenon in microorganisms. It is accelerated by the selective pressure exerted by misuse of antimicrobial agents in humans and animals [3]. Globally, inappropriate use of antibiotics is estimated to be 50% [4]. The main contributing factors for the antimicrobial resistance crisis in developing countries are a high burden of infectious diseases, irrational use of antibiotics, poor infection-control policy, substandard medicines, limited knowledge regarding AMR, misdiagnosis, and lack of laboratories for antibiotics susceptibility test [2, 5, 6].

Since AMR is a complex public health challenge, there is no single strategy that fully prevents it. Obviously, rational use of antimicrobials is the main strategy to prevent AMR. Studies reported that rational use of antimicrobials is achieved by changing the prescribing behavior and knowledge of the healthcare professionals [3, 7, 8]. It is also suggested that giving a comprehensive training and creating frequent antimicrobial resistance awareness for health students could be an effective and encouraging approach to bring rational prescribing behavior in future practitioners [9–12].

Although reports from world health organization (WHO) and other studies embraced that giving training for paramedical students on rational antimicrobial prescribing and introducing the concepts of antimicrobial stewardship into the undergraduate curricula are imperative, previous studies focused on medical students alone [11, 13–17]. Paramedical students such as Health Officer, Midwifery, Pharmacy, Nursing and Optometry that play a vital role in the prevention and promotion of antimicrobial resistance were ignored [11]. In Ethiopia, these departments have a legal and professional duty to involve in the diagnosis and management of infectious disease. Thus, this study aimed to assess knowledge and attitude about antimicrobial resistance among paramedical students even if low-priority has been given about antimicrobial resistance [18]. This is the subject of much attention to conducting this study.

## Methods

### Study design and study population

An institution based cross-sectional study was conducted at the University of Gondar, from December 2015 to March 2016. The University of Gondar is located at 730.9 km(Km) from the capital city of Ethiopia, Addis Ababa. This survey was conducted on undergraduate paramedical students at College of Medicine and Health Sciences. According to the information obtained from College of Medicine and Health Sciences assistant registrar office, there were 360 first-degree graduate students in 2015/2016 academic year in five departments including Optometry, Pharmacy, Nursing, Midwifery, and Health officer. Granting to the Ethiopian health care policy, these departments have a sound and professional obligation to take constituent in the management of infectious diseases, particularly Health officers. In the Ethiopian context, Health officers (also known as public health officers) provide comprehensive clinical outpatient and inpatient services at district health centers and they manage both the health center and woreda health offices) [19]. Even if they are a Frontline caregiver, it is conceived that there is a potential difference in their scope of practice and curricular issue. This variation may be faulted for any knowledge and attitude gap across them. The other theory was that those who had soundly knowledge antimicrobial resistance and frequent exposure to infectious disease management would have a favorable attitude regarding antimicrobial resistance. Therefore, all first-degree paramedical graduate students of each department were eligible for participation.

### Sample size determination and sampling methods

The sample size was determined using the single population proportion formula by assuming 95% confidence level, 5% margin of error, 50% proportion of poor level of knowledge and 10% non-response rate. Hence, the minimum adequate computed sample size was 423 including 10% non-response rate. Since the total target population (during the data collection period) was only 360 students, all of the students who fulfilled the eligibility criteria were considered for participation.

### Data collection procedure and tools

The data collection tool was structured questionnaire, which was developed after literature review [20–23]. The questionnaire consisted of 25 items (3 demographic, 1 source of information, 9 knowledge, and 12 attitude questions) (Additional file 1). It was mainly designed to investigate various aspects of the participants’ knowledge and attitude towards antimicrobial prescribing. The questionnaire was validated by doing pre-test on 5% of the sample before the actual data collection period. Necessary modification of the questionnaires was carried out based on the pre-test feedback. Furthermore, the reliability of the questionnaires was checked, and their Cronbach Alpha value was 0.82. The participants were approached to participate through personal communication. Then participants were invited to complete a self-administered questionnaire. The data collectors monitored the participant while filling the questionnaire so as to not use reading material and discuss with their friends. Finally, the data collectors harvested the disseminated questionnaires.

Participants’ knowledge about antimicrobial resistance was assessed using 9 questions that consisted of general knowledge about antibiotics, the cause of inappropriate use of antimicrobials, the cause of antimicrobial resistance, consequences of antimicrobial overuse and prevention strategies of antimicrobial resistance. The first 4 questions had a value of 1 or 0 (correct response had a value of ‘1′ and wrong or don’t know response had a value of ‘0′). However, the value of the last 5 questions (question 5–9) depends on the number of choices correctly chosen. Multiple responses were allowed. Each correctly chosen choice had a value of 1, and each wrongly chosen and ‘don’t know response’ had a value of 0. So the cumulative score of the last 5 questions would range from zero to 17 points for a given participant. Hence, the aggregate score for all 9 knowledge questions would range from 0 to 21 points. Participants’overall knowledge was categorized using modified Bloom’s cut-off point, as **good** if the score was between 80 and 100% (17–21 points), **moderate** if the score was between 50 and 79% (11–16 points), and **poor** if the score was less than 50% (< 11 points).

Similarly, attitude towards antimicrobial resistance was assessed using 12 questions. Responses to questions related to attitude were graded on a 3-point Likert scale, an agreement scale ranging from ‘1’ for disagree to ‘3’ for agree. The overall level of attitude was categorized using original Bloom’s cut-off point, as **positive** if the score was 80–100% (29–36 points), **neutral** if the score was 60–79% (22–28 points) and **negative** if the score was less than 60% (< 22 points). Positive attitude towards antimicrobial resistance means having a perception of that antimicrobial resistance is a catastrophic public problem and preventable if appropriate strategies are devised.

### Data processing and analysis

The collected data were checked for completeness and consistency before analysis. Incomplete questionnaires were excluded and counted as a non-response rate. Then all completed questionnaires were entered into Epidata version 3.1 and was exported to SPSS version 20 for analysis. The descriptive statistics were summarized by measure central tendency and dispersion (mean, median and range). Since the variation of knowledge and attitude across the participants’ field of study was hypothesized, a test of difference was carried out. Both knowledge and attitude scores were not normally distributed. So a nonparametric test of difference (Kruskal Wallis test and median test) at the level of significance (α) of 0.05 were employed to test the generated hypothesis. Finally, the analyzed data were organized and presented in the tabular, graphical and narrative form as per necessary.

## Results

### Characteristics of study participants

Of 360 eligible paramedical students, 90.0% (323) of them fully participated. The remaining 10.0% were not willing to participate and lack of time was the most frequent reason given not to participate. The average age of the respondents was 22.77 years ±1.52 years, ranging from 20 to 30 years. The majority of the participants were males 202 (62.5%). Regarding the participants’ distribution in terms of their field of study, the majority of the participants were from Midwifery (31.3%) and Health officer department (25.7%). Non-responders had a mean age of 22.56 years, the majority were also male 22 (59.5%) and the distribution over departments was similar 7 (19.0%).

### Participants’ knowledge about antimicrobial resistance

**Fig. 1:**
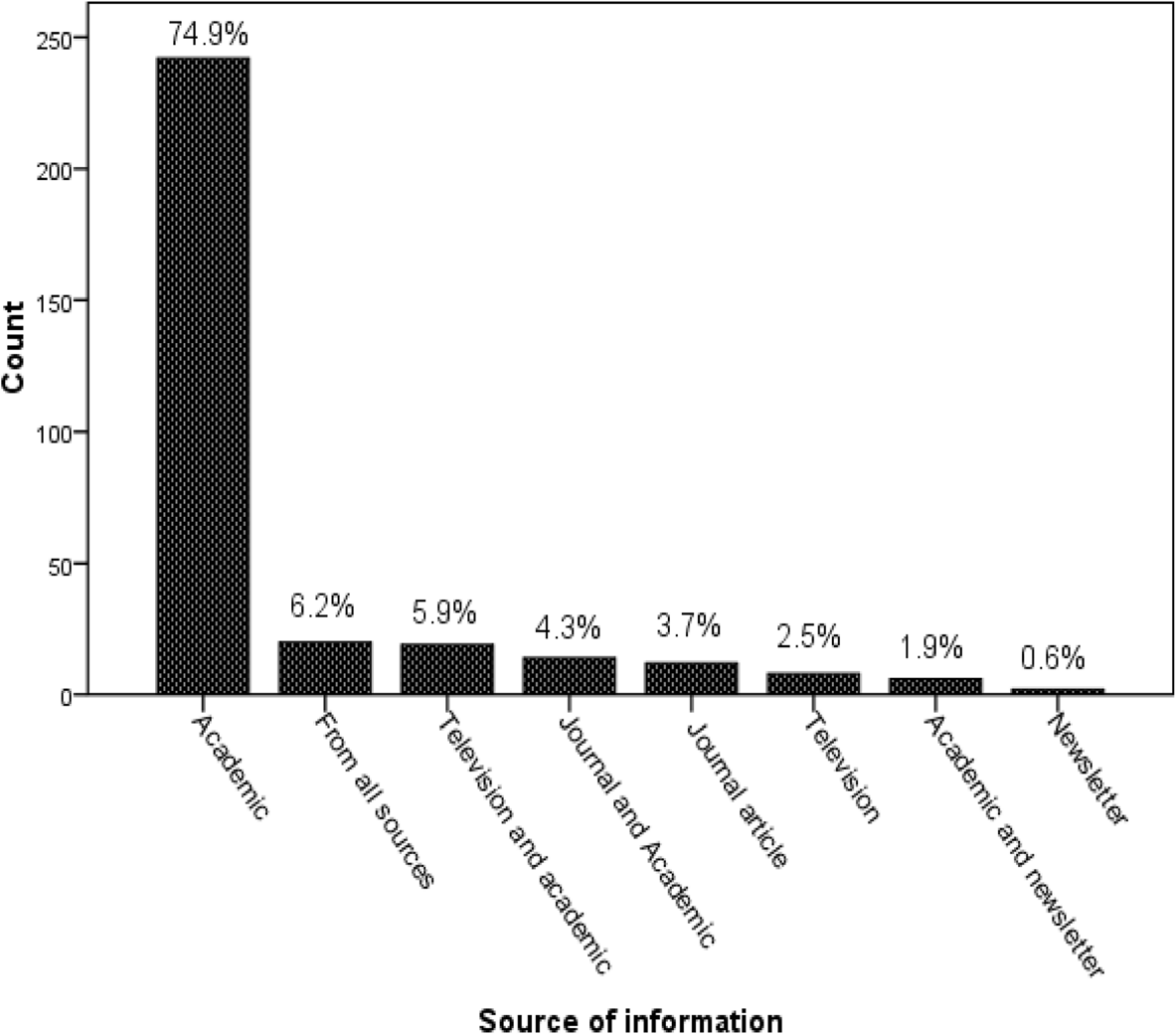
Participants’ source of information about antimicrobial resistance at University of Gondar, North West Ethiopia, 2016 (*n* = 323)

The median score of the participants’ knowledge about antimicrobial resistance was 10.0 points, ranged from 4 to 21 points. Fifty-five percent of the participants had a poor level of knowledge, followed by a moderate level of knowledge (33.1%). The majority of the study participants (82.4%) knew that frequent use of antibiotics would decrease drug efficacy. Of 323 participants, 319 (98.2%) of them conceived that inappropriate use of antibiotics puts their patients at risk. More than 50% of the study participants knew the cause of antimicrobial resistance and consequence of antibiotics overuse. However, most of them had an incorrect response to questions asked about consulting with infectious disease experts as a control strategy (65.3%), the importance of antibiotics for common cold and flu (65.0%) (Table 1 and 2). About 75% of the respondents reported that their source of information was academic courses (Fig. 1).

**Table 1:** Characteristics of study participants in College of Medicine and Health Sciences, University of Gondar, North West Ethiopia, 2016 (*n* = 323)

Variables	Frequency	Percent
Sex		
Male	202	62.5
Female	121	37.5
Department		
Midwifery	101	31.3
Health Officer	83	25.7
Nursing	75	23.2
Pharmacy	41	12.7
Optometry	23	7.1

**Table 2:** Participants’ knowledge about antimicrobial resistances among paramedical health science students at University of Gondar, North West Ethiopia, 2016 (*n* = 323)

Items	Correct	Incorrect
General knowledge about antibiotics		
1. Does inappropriate use of antibiotics put your patients at risk?	319(98.8%)	4(1.2%)
2. Does the frequent use of antibiotics will decrease its efficacy?	266(82.4%)	57(16.6%)
3. Do antibiotics speed up the recovery of common cold and flu?	113(35%)	210(65%)
4. Do antibiotics kill both viruses and bacteria?	233(72.1%)	90(27.9%)
5. Which of these do you think may promote the inappropriate use of antimicrobials?		
Poor counseling of patients	180(55.7%)	143(43.3%)
Poor skills and knowledge of prescribers	183(56.7%)	140(43.3%)
Patient Self medication	113(35%)	210(65%)
Inadequate supervision	82(25.4%)	241(74.6%)
6. Which of these factors may influence the decision to start antimicrobial therapy?		
Patient’s clinical condition	170(52.6%)	153(47.4%)
Positive microbiological results in symptomatic patients	201(62.2%)	122(37.8%)
7. Which of the following do you think are the consequences of antimicrobials overuse?		
Antimicrobial resistance	181(56%)	142(44%)
Adverse drug reactions and medication errors	193(59.8%)	130(40.2%)
Better patient outcome	315(97.5%)	8(2.5%)
8. Which of the following promote antimicrobial resistances?		
Inappropriate prescribing habits of antibiotics	163(50.5%)	160(49.5%)
Lack of effective diagnostics tools to diagnose bacterial infections	171(52.9%)	152(47.1%)
Patients self-medication	202(62.5%)	121(37.%)
Spread of bacteria in healthcare settings due to poor hygiene practices	62(19.2%)	261(80.8%)
9. Which of the following are appropriate strategies to control antimicrobial resistance?		
Targeting antimicrobial therapy to likely pathogens	149(46.1%)	174(53.9%)
Changing the attitudes of prescribers and patients	165(51.1%)	158(48.9%)
Obtaining local antimicrobial resistance profile	63(19.5%)	260(80.5%)
Consulting with infectious diseases experts	112(34.7%)	211(65.3%)
Overall level of knowledge	Frequency (%)	
Good	39(12.1)	
Moderate	107(33.1)	
Poor	177(54.8)

**Table 3:** Participants’attitude towards antimicrobial resistances among paramedical health science students at University of Gondar, North West Ethiopia, 2016 (*n* = 323)

Items	Response		
	Agree	Neutral	Disagree
1. Antimicrobial resistance will affect you and your family’s health.	293(90.7%)	19(5.9%)	11(3.4%)
2. It is necessary to give more education for final year students about antimicrobial resistance.	307(95.0%)	8(2.5%)	8(2.5%)
3. Inappropriate use of antimicrobials causes antimicrobial resistance.	304(94.1)	9(2.8%)	10(3.1%)
4. Poor infection control practices by healthcare professionals will cause the spread of antimicrobial resistance.	299(92.6%)	9(2.8%)	15(4.6%)
5. Final year students should get special training on the appropriate prescribing of antimicrobials before exit.	310(96.0%)	4(1.2%)	9(2.8%)
6. You have to follow the recommendations of your hospital antimicrobial guidelines in the future.	233(72.1%)	75(23.2%)	15(4.6%)
7. Currently, antimicrobial resistance is a major problem in the world as well as in Ethiopia.	228(70.6%)	26 (8.0%)	69(21.4%)
8. Antibiotic prescribing should be more closely controlled.	289(89.5%)	10(3.1%)	24(7.4%)
9. Dispensing antibiotics without prescription should be more closely controlled.	272(84.2%)	19(5.9)	32(9.9%)
10. People’s socioeconomic status has an effect on the risk of being affected by antibiotic resistance.	249(77.1%)	29(9.0%)	45(13.9%)
11. The consequences of antibiotic resistance will affect your future work as a health professional when caring for patients with bacterial infections.	282(87.3%)	8(2.5%)	33(10.2%)
12. Students can contribute to the work being done to control antimicrobial resistances.	284(87.9%)	21(6.5)	18 (5.6%)
Overall level of attitude	Positive	Neutral	Negative
	311(96.3%)	10(3.1%)	2(0.6%)

**Table 4:** Kruskal Wallis test to compare the participants’ knowledge and attitude score variation across their department at University of Gondar, North West Ethiopia, 2016 (*n* = 323)

	Knowledge and attitude score classified by Department						
	Department	Frequency	Median (range)	Mean rank (R)	df	Test value (H)	*P*-value
Knowledge Score	Pharmacy	41	11(5–19)	167	4	36.48	< 0.0001
	Nursing	75	9(6–16)	124			
	Health officer	83	13(4–20)	209			
	Optometry	23	12(6–19)	167			
	Midwifery	101	10(5–12)	149			
Attitude Score	Pharmacy	41	35(30–36)	180	4	17.049	0.002
	Nursing	75	34(28–36)	163			
	Health officer	83	34(29–36)	188			
	Optometry	23	34(20–36)	132			
	Midwifery	101	34(21–36)	139			
	Total	323					

The difference is significant at α =0.05(i.e. χ^2^_=_9.488)

**Table 5:** Kruskal Wallis H test for comparison of the participants’ attitude score by their level of knowledge at University of Gondar, North West Ethiopia, 2016 (*n* = 323)

	Attitude score classified by Participants’ their level of knowledge						
	Knowledge	Frequency	Mean score	Mean rank(R)	df	Test value(H)	*P*-value
Attitude Score	Good	39	35	212.4	2	32.9	< 0.0001
	Moderate	107	34.3	186.2			
	Poor	177	32.7	136.3			
	Total	323					

The difference is significant at α =0.05(i.e. χ^2^ = 9.488)

### Study participants’ attitude towards antimicrobial resistance

The median attitude score point was 34 points, ranging from 20 to 36 points. The majority of the participants 311 (96.3%) had a favorable attitude towards antimicrobial resistance. More than 70.0% of the participants agreed positively with all attitude questions that stated about the consequences of antimicrobial resistance (87.3%), the necessity of special training about antimicrobial resistance (96.0%), the cause of antimicrobial resistance (92.6%) and control strategies of antimicrobial resistance (87.9%) (Table 3).

### Comparison of the participants’ knowledge and attitude by their field of study

In Kruskal-Wallis test, there was a statistically significant knowledge difference between departments (*p* < 0.0001); with a median score of 11points (ranged from 5 to 19 points) for Pharmacy students, 9 points (range: 6–16 points) for Nursing students, 13 points (range: 4–20 points) among Health Officers students, 12points (range: 6–19 points) for Optometry students and 10 (range: 5–21 points) for Midwifery students.

Similarly, there was also a statistically significant attitude difference towards antimicrobial resistance between departments (*p* = 0.002); with a median score of 35 points (range 30–36 points) for Pharmacy students, 34 (range 28–36 points) for Nursing, 34 points (range 29–36 points) for Health Officer students, 34 points (range 20–36 points) for Optometry and 34 (range 21–36 points) for the Midwifery Department. Health officer and pharmacy students outperformed regarding knowledge and attitude concerning on antimicrobial resistance as compared to other paramedical students, respectively (Table 4).

### Comparison of the participants’ attitude scores towards antimicrobial resistance by their level of knowledge

It was also found that there was a statistically significant attitude difference towards antimicrobial resistance across the participants’ level of knowledge about antimicrobial resistance (*p* < 0.0001). Those participants with a good level of knowledge had a favorable attitude as compared to those who had a moderate and poor level of knowledge (Table 5).

## Discussion

Rational use of antimicrobials is the main strategy to prevent AMR, which is achieved by changing the prescribers’ behavior and knowledge [3, 7, 8]. In this work, it was depicted that 55 % of the participants experienced a poor knowledge about antimicrobial resistance, which was comparatively low as compared to other studies done in India, Malaysia, Portugal, Trinidad and Tobago, which reported a better understanding of antimicrobial resistance among the study participants [8, 24–26].

More than 50 % of the study participants were well informed about the effect of the frequent use of antibiotics on drug efficacy, the cause of antimicrobial resistance and the consequences of inappropriate utilization of antibiotics. Nevertheless, the bulk of the participants had a misconception about the strategies to control antimicrobial resistance, the importance of antibiotics for common cold /flu, and the essence of poor hygiene practices on the spread of bacteria in healthcare contexts. For instance, 98.8% of the participants understood that inappropriate use of antibiotics puts their patients at risk but only 35% of the participants correctly answered whether antibiotics can speed up the recovery of common cold/flu or not. This outcome was very inadequate as compared to other studies, in which 62% of students at Ahmad et al. and 95% of students at Jamshed et al. correctly answered this question [24, 26]. The target populations in Jamshed et al. and Ahmad et al. studies were only pharmacy and medical students. This might be the possible reason for the disagreement. This suggests that participants had an encouraging score on knowledge questions embedded in basic science even if they underperformed on the queries that need practical exposure.

Furthermore, misunderstanding of antibiotic indication and effectiveness was clearly noticed. Around 28% of the participants conceived that antibiotics could kill both viruses and bacteria. This result was encouraging as compared to a study conducted in Portugal, in which more than 60% of their participants stated that antibiotics should be prescribed for viral illness [25]. Such misconception may lead to high rate of inappropriate use of antibiotics, which in turn fuels the expanding antimicrobial resistance. Sadasivam et al. suggested that creating clear understanding about the therapeutic and non-therapeutic effect of antibiotic at an earlier stage of the medical education for paramedical students as well as the staff members is highly imperative [11].

A variety of resources were reported by the participants to learn about antimicrobial resistance. Since all fields included in this study have pharmacology course in their curriculum, three-fourths of the respondents reported that academic courses were their main source of information. Therefore, this implicates that giving additional emphasis regarding antimicrobial resistance, during delivering the course, might be a good opportunity to prosper the students’ knowledge and attitude.

In regard to participants’ attitude, a substantial percentage of the participants (96%) had a favorable attitude towards antimicrobial resistance (they viewed antimicrobial resistance as a public problem and preventable if appropriate strategies are devised). This result was more eminent than the studies performed in India, Trinidad, and Tobago [8, 11, 26]. Nearly three-fourths (70%) of the participants believed that antimicrobial resistance is a major problem in the universe as comfortably as in Ethiopia. This finding was lower as compared to Patel H et al. study, in which 92% of the respondents conceived that antimicrobial resistance is a local as well as a global problem [9]. Besides, the majority of the participants (82.4%) agreed that dispensing antibiotics without prescription should be more closely controlled. It was advancing as compared to another similar study, in which 65% of the participants thought that antibiotics should never be purchased as over the counter drugs [11].

Interestingly, the vast majority of the participants (96%) considered that special training on the rational use of antimicrobials and antimicrobial resistance should be given to paramedical students. This result was comparable to other studies, in which 90% of students in Abbo et al., 78% in Minen et al. and 74% in Dyar et al. pursued more education on the appropriate use of antimicrobials and proper antibiotic selection [27–29]. The potential reason for the difference might be variation target population.

In Kruskal-Wallis test, a statistically significant knowledge and attitude score difference in between the field of studies was found. Health officer and pharmacy students achieved better knowledge and attitude scores as compared to other paramedical students, respectively. There are a number of factors behind it. The main source of difference is variation in the scope of practice. Health officer and pharmacy students have frequent practical exposure to infectious disease management as compared to other health students. Some other factor is variation in their course of study. Health officer and pharmacy students took the course with higher credit per hours as compared to paramedical students. Hence, it argues that substantial efforts need to be invested in paramedical students, particularly Optometry, Midwifery, Nursing students. Furthermore, a statistically significant attitude difference towards antimicrobial resistance across the level of knowledge was noticed. Participants with a proficient level of knowledge had greater attitude rank as compared to those who possessed a moderate and poor level of cognition. This result was supported by a study done in Putrajaya, Malaysia, in which a positive correlation between mean knowledge and attitude score was found [30]. This result implicates that improving the students’ level of knowledge about antimicrobial resistance might be an approach to flourish their attitude.

Even if this work concentrated on potential target populations who play important roles in the prevention of antimicrobial resistance, the sufficiency of the sample size would not be fully addressed. It was due to the fact that the total population during the data collection period was less than the computed sample size. So generalization might be fairly limited. Another limitation was related to the design of the questionnaires. Even if the questions regarding knowledge and attitude allow the respondents to state their true thoughts without any suggestion, there is a possibility that respondents gave socially acceptable answers.

## Conclusion

Majority of the participants viewed antimicrobial resistance as a preventable public problem if appropriate strategies are invented. Nonetheless, most of them held a poor knowledge regarding antimicrobial resistance, and their knowledge and attitude significantly vary across their field of study. This result implicates that improving the students’ level of knowledge concerning the causes, consequences and controlling strategies of antimicrobial resistance might be an approach to flourish their attitude and to rationalize their antimicrobial use.

## Additional file

Additional file 1: Final edited questionnaire. (DOCX 27 kb)

## Abbreviations

AMR: Antimicrobial Resistance
Epidata: Epidemiological data
Km: Kilometer
SPSS: Statistical Package for Social Science
WHO: World Health Organization
