# Supplementary material for: Knowledge and attitude towards antimicrobial resistance among final year undergraduate paramedical students at University of Gondar, Ethiopia
Source: BMC Infect Dis. 2018 Jul 6;18:312. doi: 10.1186/s12879-018-3199-1 (PMC6035414; doi:10.1186/s12879-018-3199-1)
Supplement: Supplementary file 1 — Final edited questionnaire. (DOCX 27 kb) [file 12879_2018_3199_MOESM1_ESM.docx]

**Section I: Demographic data**

- - - 1. Age: _______________

1. Sex: Male Female
2. Department_________________________

**Section II: Questions to assess participants’ Knowledge about antimicrobial resistance**

1. **Does inappropriate use of antimicrobial put your patients at risk**?
2. Yes
3. No
4. Don’t know
5. **Are antibiotics powerful medicines to kill virus and bacteria?**
6. Yes
7. No
8. I don’t’ know
9. **Does frequent use of antibiotics decrease the efficacy of treatment?**
10. Yes
11. No
12. I don’t ’ know
13. **Do antibiotics speed up the recovery of cold, cough?**
14. Yes
15. No
16. I don’t ’ know
17. **Which of these do you think may promote the inappropriate use of antimicrobials?** (You can choose more than one)
18. Poor counseling of patients
19. Poor skills and knowledge of prescribers
20. Patient self-medication
21. Inadequate supervision
22. I don’t know
23. **Which of the following do you think are the consequences of antimicrobials overuse**? (You can choose more than one)
24. Antimicrobial resistance
25. Adverse drug reactions and medication errors
26. Better patient outcome
27. I don’t know
28. **Which of these factors may influence the decision to start antimicrobial therapy**? (You can select more than one)
29. Patient’s clinical condition
30. Positive microbiological results in symptomatic patients
31. I don’t ’ know
32. **Which of the following promote antimicrobial resistances?** (You can select more than one)
33. Inappropriate prescribing habits of antibiotics
34. Lack of effective diagnostics tools to diagnose bacterial infections
35. Patients self-medication with antibiotics without consulting health professionals
36. Spread of bacteria in healthcare settings due to poor hygiene practices
37. Don’t know
38. **Which of the following do may help to control antimicrobial resistance?** (You can select more than one)
39. Consulting with infectious diseases experts
40. Obtaining local antibiotic resistance profile
41. Targeting antimicrobial therapy to likely pathogens
42. Changing the attitudes of prescribers and patients to reduce unnecessary antibiotic usage
43. Don’t’ Know
44. **What is your source of information regarding about antimicrobial resistance?(** (You can select more than one)
    1. Academic
    2. Journal article
    3. Television
    4. Newsletter
    5. Other ____________________________________________________

**Section III: Questions to assess participants’ attitude about antimicrobial resistance**

| Items | Response | | |
| --- | --- | --- | --- |
|  | Agree | Neutral | Disagree |
| 1. Antimicrobial resistance will affect you and your family’s health. |  |  |  |
| 1. It is necessary to give more education for final year students about antimicrobial resistance. |  |  |  |
| 1. Inappropriate use of antimicrobials causes antimicrobial resistance. |  |  |  |
| 1. Poor infection control practices by healthcare professionals will cause the spread of antimicrobial resistance. |  |  |  |
| 1. Final year students should get special training on the appropriate prescribing of antimicrobials before exit. |  |  |  |
| 1. You have to follow the recommendations of your hospital antimicrobial guidelines in the future. |  |  |  |
| 1. Currently, antimicrobial resistance is a major problem in the world as well as in Ethiopia. |  |  |  |
| 1. Antibiotic prescribing should be more closely controlled. |  |  |  |
| 1. Dispensing antibiotics without prescription should be more closely controlled. |  |  |  |
| 1. People’s socioeconomic status has an effect on the risk of being affected by antibiotic resistance. |  |  |  |
| 1. The consequences of antibiotic resistance will affect your future work as a health professional when caring for patients with bacterial infections. |  |  |  |
| 1. Students can contribute to the work being done to control antimicrobial resistances. |  |  |  |
